# Immunogenic cell death in cold tumors: transforming immune-excluded tumors into immunotherapy-sensitive lesions

**DOI:** 10.3389/fphar.2026.1732407

**Published:** 2026-05-07

**Authors:** Haibo Zhao, Shiyou Dai

**Affiliations:** Qingdao Municipal Hospital, University of Health and Rehabilitation Sciences, Qingdao, China

**Keywords:** cancer immunotherapy, cold tumors, ICD, immunotherapy resistance, TME

## Abstract

Immunogenic cell death (ICD) is a distinct mode of regulated cell death capable of activating adaptive immune responses against tumor antigens, offering great promise for cancer immunotherapy. However, immune-excluded or “cold” tumors, characterized by poor immune infiltration and limited responsiveness to conventional immunotherapies, remain a major clinical challenge. ICD induction has emerged as a compelling strategy to convert these immunologically inert tumors into immunotherapy-sensitive, immune-active (“hot”) lesions by promoting dendritic cell activation, antigen presentation, and cytotoxic T-cell infiltration. This mini-review article highlights the potential of ICD-based therapeutic interventions, such as chemotherapy, radiotherapy, phototherapy, and nanoparticle-mediated delivery systems, to reprogram the immunosuppressive tumor microenvironment. By effectively converting cold tumors into immunologically responsive entities, ICD-centric approaches may significantly enhance the clinical efficacy of existing immunotherapies, highlighting the need for further translational research and personalized treatment strategies.

## 1 Introduction

Cancer immunotherapy, particularly immune checkpoint inhibitors (ICIs), has revolutionized the clinical landscape by demonstrating remarkable efficacy in various malignancies (Marei et al., 2023; Chen and Cheng, 2025; He and Xu, 2020). However, a significant proportion of patients remain refractory or develop resistance to these therapies, largely due to the immunological characteristics of the tumors themselves (Liu D. et al., 2025; Bravetti et al., 2023). Tumors can be classified immunologically into two broad categories: immunologically “hot” tumors, which harbor abundant immune cell infiltration and are typically responsive to ICIs, and immunologically “cold” tumors, which lack significant immune infiltration and often exhibit intrinsic or acquired resistance to immunotherapy (Ouyang et al., 2024; Han et al., 2024).

ICD is a specialized form of regulated cell death characterized by the emission of damage-associated molecular patterns (DAMPs), cytokines, and chemokines. These signals promote the recruitment, maturation, and activation of antigen-presenting cells (APCs), predominantly dendritic cells (DCs), thus enhancing the priming of tumor-specific cytotoxic T lymphocytes (CTLs) (Arimoto et al., 2024; Lv et al., 2025). ICD is traditionally triggered by specific chemotherapy agents, radiation therapy, targeted therapies, or physical interventions such as photothermal therapy. By activating innate and adaptive immune pathways, ICD can significantly reprogram the immunosuppressive tumor microenvironment, improving therapeutic responses and potentially achieving long-lasting anti-tumor immunity (Engelen et al., 2025; Zhou et al., 2024).

Despite these promising mechanisms, cold tumors pose a significant challenge. Such tumors, often termed immune-excluded or immune-desert, are characterized by scanty infiltration of immune cells due to physical, metabolic, and immunosuppressive barriers (Galluzzi et al., 2024; Too et al., 2021). These barriers include aberrant tumor vasculature, dense extracellular matrix, metabolic dysregulation, and accumulation of immunosuppressive cells such as regulatory T cells (Tregs), tumor-associated macrophages (TAMs), and myeloid-derived suppressor cells (MDSCs). Consequently, cold tumors exhibit resistance to ICIs and conventional immunotherapies, underscoring a critical unmet need in oncology (Dou et al., 2024).

In this mini-review, we argue that strategically leveraging ICD may provide a transformative opportunity to address these barriers. By effectively converting immune-excluded cold tumors into immunotherapy-sensitive, immune-active “hot” lesions, ICD-based therapeutic approaches could substantially expand the clinical applicability and effectiveness of cancer immunotherapies. We discuss current knowledge regarding ICD mechanisms, highlight promising preclinical and clinical strategies, and critically examine key challenges and opportunities in translating ICD into a universally applicable therapeutic approach.

## 2 Understanding the immunological landscape of cold tumors

Tumors exhibit significant heterogeneity not only in their genetic and molecular profiles but also in their immune contexture. According to their immunological characteristics, tumors are frequently classified into two main categories: “hot” and “cold” tumors. Understanding the distinct immune features of cold tumors is critical for identifying effective strategies to convert these refractory lesions into immunotherapy-sensitive states.

Cold tumors are characterized predominantly by the lack or minimal presence of infiltrating immune cells, particularly cytotoxic CD8^+^ T lymphocytes. These tumors typically present either as “immune-excluded”, where immune cells are physically restricted at the periphery and fail to penetrate the tumor parenchyma, or as immune-desert, completely lacking significant immune infiltration (Too et al., 2021; Cui et al., 2025). Such characteristics often correlate with poor prognosis and resistance to ICIs, such as anti-PD-1, anti-PD-L1, and anti-CTLA-4 therapies. In addition to the immune-excluded and immune-desert phenotypes, some cold tumors can also be viewed as an immunosuppressed subtype, in which immune cells are present but remain functionally restrained by dominant suppressive signals within the tumor microenvironment. This state is typically characterized by enrichment of regulatory T cells, M2-like tumor-associated macrophages, myeloid-derived suppressor cells, inhibitory cytokines such as TGF-β and IL-10, and metabolic suppressive circuits including adenosine accumulation. Although these tumors are not completely devoid of immune infiltration, their effector immune activity is insufficient to generate productive antitumor responses, and they therefore remain clinically resistant to immunotherapy (Liu YT. et al., 2025).

In addition to poor T-cell infiltration, cold tumors frequently exhibit low tumor mutational burden (TMB) and reduced expression of tumor-associated antigens (TAAs) or neoantigens, limiting their visibility to the immune system (Wu et al., 2024). Defective antigen presentation, such as downregulation of MHC class I molecules, β2-microglobulin mutations, or impaired antigen processing machinery (such as TAP deficiencies), further contributes to ineffective T-cell priming (Pitt et al., 2016). Moreover, dysfunctional antigen-presenting cells, particularly dendritic cells, are often scarce or fail to mature within the tumor microenvironment, preventing adequate cross-presentation of TAAs. These factors collectively weaken tumor immunogenicity and underlie the intrinsic resistance of cold tumors to immune checkpoint blockade (Bonaventura et al., 2019).

The immunological landscape of cold tumors arises from several interconnected factors. Firstly, tumor-intrinsic factors such as low TMB, limited neoantigen expression, and downregulation or loss of major histocompatibility complex (MHC) class I molecules lead to reduced tumor antigenicity. Consequently, these tumors fail to elicit robust tumor-specific T-cell responses, promoting an immunologically silent phenotype (Blair et al., 2025; Liu and Sun, 2021). Secondly, the physical and biochemical properties of the tumor microenvironment (TME) profoundly contribute to immune exclusion. Dense extracellular matrix deposition and increased tumor-associated fibrosis impede T-cell trafficking and infiltration. Aberrant angiogenesis and dysfunctional tumor vasculature further restrict immune cell entry and create hypoxic and nutrient-depleted regions, promoting immunosuppressive conditions that impair T-cell function and survival (Lv et al., 2024; Lamplugh and Fan, 2021). Thirdly, the immunosuppressive microenvironment is reinforced by an abundance of inhibitory immune cells, such as Tregs, TAMs with M2 phenotype, and MDSCs. These cells release immunosuppressive factors including transforming growth factor-beta (TGF-β), interleukin-10 (IL-10), vascular endothelial growth factor (VEGF), and metabolites such as adenosine and kynurenine. These mediators collectively create a hostile milieu that suppresses effector T-cell activity and maturation of DCs, further exacerbating immune exclusion and tumor progression (Tie et al., 2022; Jennings et al., 2021).

Collectively, these multifaceted tumor-intrinsic and extrinsic mechanisms contribute to the formation of cold tumors, ultimately leading to resistance against conventional immunotherapies. Therefore, converting the immunological landscape of these tumors by overcoming physical, metabolic, and immunosuppressive barriers represents a vital and promising therapeutic goal. Leveraging therapeutic strategies that induce ICD, which can directly alter antigenicity, enhance immunological visibility, and modify the immunosuppressive TME, holds potential to reshape cold tumors into immune-active, “hot” lesions, thereby enabling responsiveness to immunotherapy.

Importantly, the responsiveness of different cold tumor subtypes to ICD induction is unlikely to be uniform. Immune-desert tumors, which lack effective priming and baseline immune infiltration, may require ICD to first enhance antigen release, dendritic-cell recruitment, and T-cell priming. Immune-excluded tumors may derive greater benefit from ICD when it is combined with strategies that overcome stromal and vascular barriers to facilitate intratumoral T-cell penetration. By contrast, immunosuppressed tumors may be more likely to benefit from ICD when it is paired with approaches that relieve dominant suppressive pathways, such as TGF-β-, myeloid-, or adenosine-targeted interventions. Therefore, recognizing cold tumors as biologically distinct immune states may improve the clinical guidance value of ICD-based therapeutic design (Ji et al., 2025).

## 3 Immunogenic cell death: mechanisms and potential for tumor microenvironment modulation

ICD represents a unique and therapeutically relevant form of regulated cell death characterized by its capacity to activate potent adaptive immune responses against tumor-associated antigens. Unlike conventional forms of apoptosis, which typically proceed silently without eliciting significant immune responses, ICD is marked by the coordinated release of multiple immunostimulatory signals (Galluzzi et al., 2024). These signals profoundly modulate the local TME, transitioning it from an immunosuppressive niche into an immunologically active site capable of recruiting, activating, and sustaining antitumor immune cells (Fucikova et al., 2020).

ICD is mechanistically defined by the emission of DAMPs from dying cells, which act as critical danger signals recognized by pattern recognition receptors (PRRs) on APCs, primarily DCs. Prominent DAMPs associated with ICD include surface-exposed calreticulin (CALR), extracellular adenosine triphosphate (ATP), high-mobility group box 1 protein (HMGB1), annexin A1 (ANXA1), and heat shock proteins (HSP70 and HSP90) (Fucikova et al., 2020; Fucikova et al., 2014). Specifically, CALR translocates from the endoplasmic reticulum (ER) to the plasma membrane surface during ICD, serving as a crucial “eat-me” signal for DCs, thus facilitating the phagocytosis of dying tumor cells. Extracellular ATP further amplifies this process by stimulating the purinergic receptor P2RX7 on DCs, triggering inflammasome activation and subsequent secretion of proinflammatory cytokines, notably IL-1β and IL-18. HMGB1 released from the nucleus binds Toll-like receptor 4 (TLR4) on DCs, promoting their maturation and antigen-presenting capabilities (Jiang et al., 2025; Chang et al., 2023).

Beyond DAMP release, several cellular stress pathways critically shape the induction of ICD. ER stress plays a central role by promoting the surface exposure of calreticulin and other chaperones, thereby serving as ‘eat-me’ signals for dendritic cells (Galluzzi et al., 2024). Autophagy contributes to ICD by facilitating ATP release into the extracellular space, which activates purinergic signaling and inflammasome pathways. In addition, the integrated stress response (ISR), characterized by phosphorylation of eIF2α and translational reprogramming, enhances the expression of stress-related molecules that amplify immunostimulatory signaling (Fucikova et al., 2020; Jiang et al., 2025). Together, these processes not only determine the quality and magnitude of ICD but also synergize to establish an inflammatory tumor microenvironment that potentiates adaptive immune responses.

Mechanistically, ER stress and ISR are closely linked in ICD through the PERK-eIF2α signaling axis. Upon unresolved endoplasmic reticulum stress, PERK is activated and phosphorylates eIF2α, which transiently attenuates global protein translation while selectively promoting stress-adaptive transcriptional programs. In the context of ICD, this pathway facilitates premortem stress signaling associated with calreticulin exposure and enhances the immunogenicity of dying tumor cells. Autophagy acts in parallel and cooperates with ER stress/ISR signaling by sustaining ATP secretion from stressed or dying cells, thereby promoting purinergic signaling, inflammasome activation, and dendritic-cell recruitment. Thus, ER stress-PERK-eIF2α signaling primarily contributes to the qualitative conversion of tumor cell death into an immunogenic state, whereas autophagy helps ensure the release of key immunostimulatory mediators. The coordinated activation of these pathways is therefore a major determinant of whether tumor cell stress culminates in *bona fide* ICD rather than non-immunogenic cell death (Zhao et al., 2025).

**FIGURE 1:**
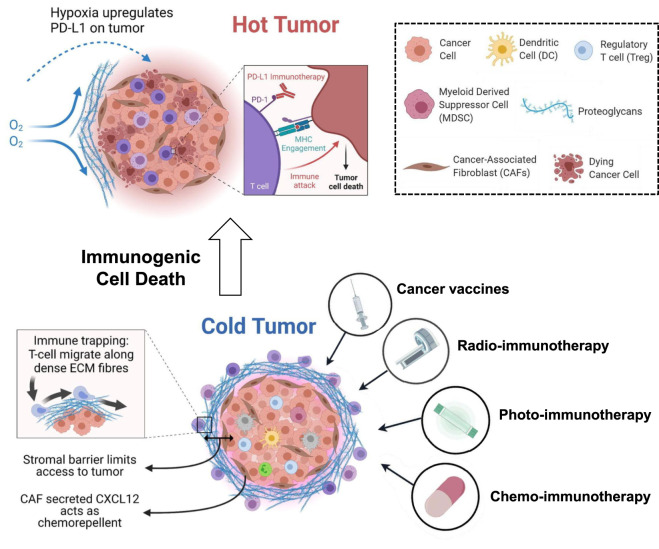
Schematic illustration of ICD-induced DAMP release (CALR, ATP, HMGB1), which activates dendritic cells and promotes T cell infiltration, thereby remodeling the immunosuppressive tumor microenvironment and converting cold tumors into immunotherapy-responsive hot tumors.

Figure 1 provides a schematic overview of the ICD process, while the text below further summarizes the key mechanistic links among ER stress, autophagy, and ISR signaling that support immunogenic DAMP emission and downstream antitumor immunity. Although the figure primarily illustrates the ICD process, it should be interpreted in the broader context of the tumor immune landscape. In cold tumors, limited immune infiltration, aberrant vasculature, and an immunosuppressive cytokine milieu create barriers to effective antitumor immunity. By contrast, hot tumors are characterized by abundant T-cell infiltration, normalized vascular networks, and a pro-inflammatory cytokine environment. ICD induction serves as a critical bridge, helping to remodel the cold tumor microenvironment toward a hot, immune-responsive state. ICD induction by therapeutic interventions results in tumor cell death accompanied by the coordinated release of DAMPs such as CALR, ATP, and HMGB1. These signals facilitate DC recruitment, maturation, and antigen presentation, ultimately leading to the priming and infiltration of cytotoxic CD8^+^ T lymphocytes into the previously immune-excluded tumor microenvironment. As illustrated, this series of events effectively transforms the immunosuppressive tumor niche into an immune-active environment, thereby sensitizing tumors to subsequent immunotherapeutic interventions.

ICD-inducing therapeutic strategies encompass various agents and modalities, including certain chemotherapeutic drugs (anthracyclines like doxorubicin, oxaliplatin, paclitaxel), targeted anticancer therapies (crizotinib, cabozantinib), radiation therapy, photodynamic and photothermal therapy, and oncolytic viruses. These treatments induce potent ICD by generating intense cellular stress through DNA damage, disruption of microtubule dynamics, or severe oxidative stress, thereby triggering the characteristic immunogenic signaling cascade (Zhai et al., 2023; Wang et al., 2024).

Beyond directly stimulating adaptive immunity, ICD profoundly reshapes the tumor microenvironment to become more favorable for antitumor immune responses. It increases tumor antigenicity by releasing neoantigens and enhanced antigen processing via APCs. Simultaneously, it increases adjuvanticity by providing powerful co-stimulatory and inflammatory signals, recruiting immune effector cells into previously immunologically inert tumor regions. This dual enhancement of antigenicity and adjuvanticity drives the maturation and infiltration of DCs and the subsequent priming and activation of tumor-specific CTLs (Mardi et al., 2022; Hernández et al., 2021). Collectively, these alterations in the TME counteract immunosuppressive conditions, reduce immune evasion mechanisms, and significantly improve the likelihood of clinical response to immunotherapies, such as immune checkpoint blockade. Given these powerful effects, strategically leveraging ICD represents a highly promising approach to transforming cold, immune-excluded tumors into immunologically “hot” therapeutically responsive lesions.

## 4 Strategies and clinical translation of ICD-based immunotherapies in immune-excluded tumors

Given the challenges posed by “cold” tumors, innovative therapeutic strategies designed to induce or enhance ICD have become a promising avenue for transforming these tumors into “hot” lesions responsive to immunotherapies. Several cutting-edge approaches have emerged to enhance ICD and remodel the TME, primarily focused on improving antigenicity, adjuvanticity, immune infiltration, and reducing immunosuppression. Importantly, many of these strategies have already entered clinical testing, and representative trials are discussed alongside each approach to highlight the current evidence base and translational progress.

Clinical combinations of ICD-inducing chemotherapies such as oxaliplatin, doxorubicin, cyclophosphamide, and paclitaxel with ICIs (anti-PD-1/PD-L1 and anti-CTLA-4) have demonstrated encouraging efficacy in refractory tumors. For example, the combination of oxaliplatin-based chemotherapy with pembrolizumab has been evaluated in metastatic colorectal cancer (NCT02375672) and metastatic pancreatic cancer (NCT04181645), aiming to potentiate immunotherapy efficacy via ICD-driven immune modulation (Herting et al., 2021; Cui et al., 2020). Similarly, the TONIC trial (NCT02499367) utilized a short induction of chemotherapy (including doxorubicin) to prime triple-negative breast cancer patients before nivolumab, showing improved tumor immune infiltration and promising response rates (Hudeček et al., 2020).

### 4.1 Combination therapies with ICD-Inducing chemotherapeutics and immunotherapy

Combination treatments integrating conventional chemotherapy with ICIs represent a clinically viable strategy for enhancing ICD. Chemotherapeutics such as anthracyclines (doxorubicin), oxaliplatin, cyclophosphamide, paclitaxel, and proteasome inhibitors (bortezomib) can trigger robust ICD via DNA damage, ER stress, and autophagy induction, releasing abundant immunostimulatory DAMPs (Zhai et al., 2023; Yerragopu et al., 2023). Subsequent administration of ICIs targeting PD-1/PD-L1 or CTLA-4 enhances the immune-activating effects initiated by ICD, promoting the expansion and function of tumor-specific cytotoxic T cells (Kepp et al., 2019). Preclinical and clinical studies have demonstrated that such combinatorial strategies markedly improve outcomes, particularly in tumors previously considered unresponsive to immunotherapy.

### 4.2 Radiotherapy as an ICD inducer and immunomodulator

Radiotherapy, especially focal radiation delivered at precise doses, effectively induces ICD through direct DNA damage and induction of oxidative stress, subsequently promoting robust adaptive immune responses (Sheikh and Curran, 2025). Recent developments in radiotherapy strategies, such as stereotactic body radiotherapy (SBRT), carbon-ion radiotherapy, and proton beam therapy, demonstrate enhanced ability to initiate ICD by triggering pronounced inflammatory responses within tumors (Altorki et al., 2021; Vaes et al., 2021; Zhu et al., 2022). Furthermore, radiation-induced ICD can synergize effectively with immunotherapy, providing additional co-stimulatory signals necessary to activate and sustain cytotoxic T-cell responses (Kroemer et al., 2024). Optimizing radiation dose, fractionation schedules, and timing relative to ICIs could significantly amplify the immune response in previously refractory tumors.

Consistent with this rationale, clinical studies combining radiotherapy with PD-1/PD-L1 or CTLA-4 blockade have reported improved response signals in otherwise immunotherapy-insensitive settings, supporting ICD-driven immune modulation as a plausible mechanism. Radiotherapy-induced ICD combined with ICIs has provided promising clinical outcomes, particularly in tumors traditionally insensitive to immunotherapy. A landmark study (PEMBRO-RT, NCT02492568) combining pembrolizumab with radiotherapy in advanced non-small cell lung cancer (NSCLC) patients demonstrated increased overall response rates and prolonged progression-free survival, attributed to ICD-driven immune modulation of the tumor microenvironment (Theelen et al., 2019). Likewise, trials such as the RADVAX study (NCT01497808) in prostate cancer, combining ipilimumab with radiotherapy, revealed immunological changes consistent with ICD mechanisms, further validating this therapeutic concept (Maity et al., 2021).

### 4.3 Nanoparticle-based delivery systems to improve ICD

Nanomedicine provides a versatile toolbox to enhance tumor immunogenicity and overcome the physical and biochemical barriers that typify immune-excluded tumors. Beyond improving intratumoral exposure of ICD inducers, nanoplatforms can (i) co-deliver antigens and immune adjuvants to amplify dendritic-cell activation and T-cell priming; (ii) integrate stimulus-responsive or energy-converting components (such as PDT/PTT/SDT-enabled ROS/heat generation) to intensify ER stress and DAMP emission; and (iii) remodel hostile microenvironmental features such as hypoxia/acidity to facilitate immune infiltration and rational combination with ICIs. (Qi et al., 2024; Hiep Tran and Thu Phuong Tran, 2024). As summarized in a recent comprehensive review, these functions collectively increase tumor immunogenicity via targeted antigen delivery, ICD induction, and microenvironmental reprogramming (Dogheim et al., 2024).

Clinically, nanoplatforms enable spatiotemporal control over ICD triggers and adjuvant release, which is particularly relevant for immune-excluded tumors with high stromal barriers. Several nano-formulations are now being tested clinically, providing early translational evidence for this approach. Innovative nanoparticle formulations aiming to deliver ICD-inducing agents specifically to tumor sites are entering clinical evaluation. For example, CRLX101, a camptothecin-based nanoparticle therapy inducing ICD via potent DNA damage and ER stress, has undergone clinical trials in combination with pembrolizumab for advanced solid tumors, including ovarian and colorectal cancers (NCT02769962). This strategy aims to maximize therapeutic effects through enhanced tumor-specific ICD induction while minimizing systemic toxicities (Schmidt et al., 2020). In parallel, nanoparticles encapsulating immune agonists can spatially confine adjuvant signals within tumors and draining lymph nodes, thereby strengthening ICD-driven adjuvanticity and subsequent T-cell expansion (Ma P. et al., 2023; Gong et al., 2019). Additionally, nanoparticles engineered to respond to specific TME conditions—such as hypoxia, acidity, or proteolytic enzymes—can further optimize controlled drug release, providing a tailored therapeutic response (Liu et al., 2024).

### 4.4 Localized ICD-Inducing approaches: phototherapy, ablation, and oncolytic viruses

Local interventions, such as photodynamic therapy (PDT) and photothermal therapy (PTT), leverage external energy sources to precisely induce ICD in targeted tumor regions. Because immune-excluded tumors are often characterized by spatial barriers, localized ICD induction can create an *in situ* inflammatory focus that promotes dendritic-cell activation and subsequent T-cell recruitment across the stromal boundary. Oncolytic virotherapy represents another promising ICD-inducing strategy. Talimogene laherparepvec (T-VEC), an FDA-approved oncolytic virus for advanced melanoma, triggers ICD via direct tumor lysis and immune activation. Clinical studies combining T-VEC with pembrolizumab (KEYNOTE-034, NCT02263508) reported significantly improved clinical responses compared to historical outcomes with monotherapy, highlighting the clinical value of combining virotherapy-induced ICD with ICIs (Kjeldsen et al., 2021; Chesney et al., 2022).

Localized approaches, including intratumoral administration of ICD-inducing peptides, also show promise. For instance, LTX-315, an oncolytic peptide designed to induce robust ICD, has entered phase I/II trials (NCT01986426), demonstrating encouraging immunological responses and tumor regression, thus supporting further exploration of localized ICD induction combined with systemic immunotherapy (Jebsen et al., 2019).

These methods generate localized oxidative stress, hyperthermia, and necrotic cell death, releasing DAMPs and initiating robust immune responses (Fang et al., 2025; Alzeibak et al., 2021). Additionally, minimally invasive physical ablation techniques (microwave ablation, high-intensity focused ultrasound) offer similar benefits by rapidly inducing localized cell death and subsequent immune activation (Yu et al., 2014). Oncolytic viruses engineered to selectively replicate within tumor cells can also potently induce ICD. Genetic modifications enhancing viral immunogenicity or encoding immune-activating cytokines (GM-CSF, IL-12, IFN-α) further amplify antitumor immunity (Ma R. et al., 2023).

### 4.5 Personalized ICD induction based on tumor microenvironment profiling

Considering tumor heterogeneity, personalized strategies guided by precise TME profiling represent a forward-looking approach. Advanced genomic, transcriptomic, and proteomic analyses, along with spatial characterization of the TME using novel imaging technologies, could enable the identification of optimal ICD-inducing regimens for individual patients (Reijers et al., 2022). By predicting ICD responsiveness through specific biomarkers—such as expression of ER-stress-related genes, autophagy pathways, or presence of immune-inhibitory cells—clinicians could tailor ICD induction therapies, including optimal selection of drugs, doses, schedules, and combinations with immunotherapy, thus maximizing clinical efficacy. In parallel, embedding ICD-related biomarkers (such as CALR exposure, HMGB1/ATP release signatures, ER-stress/autophagy programs, and spatial immune profiling) into ongoing trials may help explain heterogeneous responses and guide rational selection of priming regimens.

A concise biomarker framework may further support personalized ICD induction. Among the most widely recognized ICD-associated markers, surface-exposed CALR reflects pro-phagocytic signaling, extracellular ATP indicates active danger signaling and inflammasome engagement, and released HMGB1 supports dendritic-cell maturation and antigen processing. In parallel, TLR4 competence may influence the sensing of HMGB1-dependent immunogenic signals, whereas preserved or therapy-induced MHC-I expression is relevant to the effective presentation of tumor antigens to cytotoxic T cells. Together, these markers may serve as practical indicators for monitoring ICD induction and estimating whether tumor cell stress is likely to translate into productive antitumor immunity (Liu et al., 2026).

### 4.6 Critical comparison of ICD-inducing strategies and current translational bottlenecks

Although multiple therapeutic modalities can induce ICD, their translational value is not equivalent across tumor settings (Liu et al., 2026; Catanzaro et al., 2025). Chemotherapy and radiotherapy remain the most clinically accessible ICD inducers and have relatively clear integration paths with immune checkpoint inhibitors, particularly in tumors such as non-small cell lung cancer, triple-negative breast cancer, and gastrointestinal malignancies. However, their immunostimulatory effects are highly context-dependent and may be offset by systemic toxicity, lymphodepletion, or suboptimal treatment scheduling. By contrast, nanoparticle-based platforms and localized approaches such as phototherapy or ablation offer improved spatial control and reduced off-target exposure, but their broader clinical implementation is limited by manufacturing complexity, delivery heterogeneity, and regulatory barriers. Oncolytic viruses are particularly attractive for immunologically inert lesions because they combine direct oncolysis with inflammatory reprogramming, yet their efficacy can be constrained by antiviral immunity, intratumoral delivery challenges, and variable replication efficiency across tumor types. Recent studies also indicate that resistance to ICD-based strategies may arise from defective antigen presentation, persistent TGF-β- or adenosine-dominant immunosuppression, stromal exclusion of T cells, and insufficient dendritic-cell priming. Therefore, the key clinical bottleneck is not simply whether a treatment can induce ICD, but whether ICD can be effectively coupled to downstream antigen presentation, T-cell recruitment, and immune checkpoint release in a tumor-type-specific manner.

### 4.7 Safety considerations and clinical management of toxicities in ICD-based combinations

ICD-based regimens are most often implemented as combinations (such as ICD-inducing chemotherapy, radiotherapy, nanomedicine, or local therapies plus immune checkpoint inhibitors), and their safety profile therefore reflects both modality-specific toxicities and immune-related adverse events (irAEs). Cytotoxic or ICD-inducing agents may cause predictable treatment-related toxicities depending on the platform (myelosuppression and gastrointestinal toxicity with chemotherapy; tissue inflammation with radiotherapy; photosensitivity or local inflammatory reactions with PDT/PTT; procedure-related complications after ablation; flu-like symptoms and local reactions with oncolytic virotherapy). When layered with ICIs, the principal additional concern is the potential amplification of irAEs across organ systems, underscoring the need for proactive monitoring and timely intervention (Brahmer et al., 2018).

In clinical practice, toxicity management should follow established irAE frameworks that emphasize early recognition, grading, and stepwise treatment escalation. In general, mild events may be managed with close monitoring, while persistent or moderate events typically warrant treatment interruption and supportive care, with systemic corticosteroids considered when clinically indicated; severe or life-threatening events require urgent multidisciplinary management and immunosuppression per guideline recommendations. These principles are particularly relevant for ICD-priming trials that intentionally increase tumor inflammation (chemotherapy priming as in TONIC or radiotherapy priming as in PEMBRO-RT), where careful scheduling and surveillance are essential to maintain the therapeutic window while preserving tolerability (Brahmer et al., 2021).

To improve safety without compromising immunogenic efficacy, optimization can be implemented at multiple levels: (i) rational dose and fractionation design (especially for radiotherapy) and avoidance of unnecessary overlap of peak inflammatory phases; (ii) use of tumor-targeted delivery (including nanomedicine or localized approaches) to increase intratumoral exposure while reducing systemic burden; and (iii) patient selection and baseline risk assessment (such as pre-existing autoimmune conditions, organ reserve, prior treatment history), ideally coupled with ICD-relevant and immune-monitoring biomarkers to guide continuation, de-escalation, or rechallenge. Collectively, aligning ICD induction with guideline-based toxicity management and precision scheduling will be critical for broad clinical adoption of ICD-based immunotherapy strategies (Powles et al., 2021).

Collectively, the above strategies illustrate that ICD induction is increasingly being translated from mechanistic rationale into clinical testing across multiple tumor types. Emerging trial signals suggest improved immune infiltration and response rates when ICD-priming is appropriately sequenced with immunotherapies, supporting continued optimization of patient selection, dosing, and scheduling. Given that ICD priming often intensifies local and systemic inflammation, we further summarize safety considerations and guideline-aligned toxicity management for ICD-based combinations. A summary of representative therapeutic approaches, their mechanisms of ICD induction, and relevant clinical trials is provided (Table 1).

**TABLE 1:** Summary of ICD-Inducing therapeutic approaches for converting cold tumors into immunotherapy-sensitive lesions.

Therapeutic approach	Representative agents/Techniques	Mechanisms of ICD induction	Clinical trial examples (NCT number)
Chemotherapy-induced ICD	Doxorubicin, oxaliplatin, cyclophosphamide, paclitaxel, bortezomib	DNA damage, ER stress, autophagy, release of DAMPs (CALR, HMGB1, ATP)	NCT02375672, NCT04181645, NCT02499367 (TONIC)
Radiotherapy-induced ICD	Photon radiation, carbon-ion therapy, proton beam therapy	DNA damage, oxidative stress, cytokine release	NCT02492568 (PEMBRO-RT), NCT01497808 (RADVAX)
Phototherapy-based ICD	Photodynamic therapy (PDT), photothermal therapy (PTT)	ROS generation, thermal ablation, local inflammation, DAMP release	Ongoing preclinical studies
Oncolytic virus-based ICD	Talimogene laherparepvec (T-VEC), engineered adenoviruses	Direct tumor lysis, immune activation, cytokine induction	NCT02263508 (KEYNOTE-034), NCT01986426 (LTX-315)
Nanoparticle-mediated ICD	CRLX101 (camptothecin-based nanoparticles), other drug-loaded nanoparticles	Enhanced drug delivery, tumor-specific induction of DNA damage, ER stress, and autophagy	NCT02769962
Localized ablation techniques inducing ICD	Microwave ablation, high-intensity focused ultrasound (HIFU)	Rapid necrotic cell death, release of immunogenic molecules and inflammatory cytokines	Preclinical and early-phase clinical studies ongoing

## 5 Conclusion

ICD represents an innovative and clinically meaningful strategy for addressing the significant therapeutic challenge posed by “cold” tumors. Unlike conventional forms of apoptosis, ICD uniquely stimulates robust adaptive immune responses, thus offering a promising approach for converting resistant, immunologically inert tumors into immunotherapy-sensitive, immune-active lesions. Through mechanisms involving the release of DAMPs, activation of APCs, and priming of cytotoxic T cells, ICD induction can effectively transform the immunosuppressive TME, enabling improved therapeutic outcomes.

Despite the considerable potential demonstrated by preclinical and clinical studies, several critical challenges persist, including tumor heterogeneity, immunosuppressive TME barriers, variable responsiveness to ICD-inducing agents, and limitations in currently available biomarkers and preclinical models. Addressing these challenges requires continued translational research, particularly in biomarker discovery, personalized therapy optimization, and development of improved immune monitoring technologies.

Looking forward, further advancement in precision oncology, including tailored treatment strategies guided by tumor-specific molecular and immunological characteristics, will significantly enhance ICD’s clinical efficacy. Emerging combination therapies integrating next-generation immunotherapeutic agents, novel immune modulators, and advanced nanoparticle delivery technologies are poised to further amplify ICD-mediated antitumor immunity, broadening the scope of effective cancer immunotherapy. Ultimately, harnessing the full therapeutic potential of ICD will require multidisciplinary collaboration among immunologists, oncologists, pharmacologists, bioengineers, and clinical researchers. Continued investment in clinical research, biomarker discovery, technology development, and patient-specific therapeutic approaches will be essential to firmly establish ICD-based strategies as a cornerstone of modern immunotherapy. Because the immunologically ‘cold’ tumor phenotype occurs across diverse cancer types, ICD-based strategies hold pan-tumor significance for overcoming immune resistance and enhancing the efficacy of immunotherapy. Such efforts promise to significantly expand the population of cancer patients benefiting from immunotherapy, transforming clinical outcomes for patients previously facing limited therapeutic options.
